# Low enrollment and high treatment success in children with drug-resistant tuberculosis in Ethiopia: A ten years national retrospective cohort study

**DOI:** 10.1371/journal.pone.0229284

**Published:** 2020-02-26

**Authors:** Habteyes Hailu Tola, Kourosh Holakouie-Naieni, Mohammad Ali Mansournia, Mehdi Yaseri, Ephrem Tesfaye, Zemedu Mahamed, Million Molla Sisay

**Affiliations:** 1 Department of Epidemiology and Biostatistics, Tehran University of Medical Sciences (TUMS), School of Public Health, Tehran, Iran; 2 Tuberculosis/HIV Research Directorate, Ethiopian Public Health Institute, Addis Ababa, Ethiopia; 3 Saint Peter’s Specialized Hospital, Research and Evidence Generation Directorate, Addis Ababa, Ethiopia; Institute of Social and Preventive Medicine, SWITZERLAND

## Abstract

**Background:**

Limited evidence exists on the treatment outcome and factors that are associated with the duration from the initiation of treatment to death or treatment failure in children with drug resistant tuberculosis (DR-TB). Thus, we aimed to determine the proportion of treatment enrollment, status of treatment outcome and determine factors that are associated with the duration from treatment initiation to death or treatment failure in children treated for DR-TB in Ethiopia.

**Methods:**

We conducted a retrospective cohort study in children younger than 15 years old who were treated for DR-TB from February 2009 to February 2019 in Ethiopia. We collected data on socio-demographic and clinical characteristics from clinical charts, registration books and laboratory result reports on 155 children. Proportion of enrollment to the treatment was calculated by dividing the total number of children who were receiving the treatment by the total number of DR-TB patients treated during the specified years. We used Cox proportional hazard models to determine factors that were associated with the duration from the beginning of the treatment to death or treatment failure. Data was analyzed using STATA version 14.

**Results:**

Of the 3,478 DR-TB patients enrolled into the treatment and fulfilling our inclusion criteria during the past ten years, 155 (4.5%) were children. Of the 155 children, 75 (48.4%) completed the treatment and 51 (32.9%) were cured. Furthermore, 18 (11.6%) children were died, seven (4.5%) lost to follow up and treatment of four (2.6%) children was failed. The overall treatment success was 126 (81.3%). Age younger than 5 years old [*Adjusted Hazard Ratio (AHR) = 3*.*2*, *95%CI (1*.*2–8*.*3)*], HIV sero-reactivity [*AHR = 5*.*3*, *95%CI (1*.*8–14*.*9)*] and being anemic [*AHR = 4*.*3*, *95%CI (1*.*8–10*.*3)*] were significantly associated with the duration from the enrollment into the treatment to death or treatment failure.

**Conclusion:**

In this study, the proportion of children enrolled into DR-TB treatment was lower than the proportion of adults enrolled to the treatment (4.5% in children versus 95.5% in adults) in last ten years. Our findings also suggest that children with DR-TB can be successfully treated with standardized long term regimen. Further prospective cohort study is required to investigate factors contributing to death or treatment failure.

## Background

Tuberculosis (TB) is among the top 10 causes of death globally, and an estimated 10 million new cases and 1.6 million deaths occurred in 2017 [1]. Multidrug resistant (MDR) and extensively drug resistant (XDR) TB are becoming a global public health threat due to difficulty in performing drug susceptibility test (DST) and poor treatment response to existing medication [1]. An estimated 558,000 new MDR-TB/Rifampin Resistant TB cases occurred worldwide in 2017 [1]. MDR-TB refers to a *Mycobacterium tuberculosis* strain resistant to at least isoniazid and rifampin [2]. Rifampin resistant TB (RR-TB) is defined as a *M*. *tuberculosis* strain that is rifampin mono/poly resistant on the basis of drug susceptibility test (DST) result [2]. In this study, we defined DR-TB as *M*. *tuberculosis* resistant to any of the first line drugs that are used to treat drug susceptible TB (DS-TB) and eligible for treatment with MDR-TB regimens.

Although pediatric TB has been gaining attention in recent years, it is one of the most neglected public health problems [3,4] because low proportion of children are diagnosed and enrolled to the treatment compared to adults. TB in children accounts about 15–20% of global TB burden [4–6], and it is among the top ten causes of mortality in under-5 children worldwide [6]. Moreover, a global estimate indicated that about half a million TB cases occur per year in children younger than 15 years [5].

Diagnosis of DR-TB is the most challenging in resource limited countries due to limited laboratory infrastructure for DST and the long duration required for laboratory confirmation [4–6]. Even diagnosis of DR-TB in children is more difficult and it is rarely bacteriologically confirmed due to paucibacillary nature of the disease and the challenges in obtaining appropriate specimens [7]. A recent estimate indicates that about 25,000 to 32,000 of children develop DR-TB annually, which accounts for 3% of TB cases in children in the world [7]. Children are also at greater risk of contracting DR-TB from adult cases due to their biological and social vulnerability. The burden of the disease is also overlooked in this group of population due to difficulty in diagnosis and bacteriological confirmation [4,8]. Moreover, despite the number of children contracting DR-TB is increasing globally, still small proportion of children are detected and enrolled to the treatment [4].

Treating DR-TB cases is more expensive and difficult than treating drug-susceptible TB cases [9,10]. Moreover, second line drugs (SLDs) used for the treatment of DR-TB are more toxic and less effective than the first line drugs [9–11]. The number of drugs and duration of treatment required for the successful treatment outcome in children infected with DR-TB is unknown because of the lack of strong evidence from different countries on statistically optimum sample size [12,13]. Recently, there is some evidence on the magnitude and status of treatment outcomes of DR-TB in children [14–25]. This evidence indicates that the current treatment increases the likelihood of treatment success and has the potential of decreasing the mortality rate [14–25]. For instance, a study reported from Peru [25] shows that 77.2% of children achieved cure or probable cure. A study reported from South Africa [26] also indicated that 68% of children treated for DR-TB had the probability of curing from the disease. A systematic review and meta-analysis studies reported on children with DR-TB is also indicated considerable treatment success [12,13]. However, due to data limitation in certain settings, the World Health Organization (WHO) recommends that DR-TB treatment in children should be based on expert opinion or extrapolation of adult treatment guidelines wherever evidence on children is unavailable [27].

The current global treatment success of DR-TB is low, and only 55% of DR-TB cases were successfully treated in 2017 [1]. A recently reported review studies also indicated low (61% and 64%) DR-TB treatment success in adults [28,29]. However, DR-TB treatment success in children is relatively higher than in adults [12,13]. For instance, a meta-analysis study pooled data on 975 children with DR-TB has reported 78% treatment success [13]. Another meta-analysis study which included 315 children with DR-TB also reported 82% of treatment success [12] which is relatively higher than the previous studies’ reports on adults (61% and 64%) [28,29].

Ethiopia is among the 30 countries with high TB, TB/HIV and MDR-TB burden [1]. Although, earlier studies have indicated high treatment success of DR-TB in all age group patients in Ethiopia [30,31], there is no national or regional level report on the burden and treatment outcome status in children infected with DR-TB. However, the earlier study [31] included only 5% of patients who were younger than 18 years old and the youngest participant was 8 years old. In addition, there is limited evidence on the magnitude of children infected with DR-TB and enrolled to the treatment in the country. Therefore, we aimed to determine the proportion of children diagnosed and enrolled to the treatment in the study period, treatment outcome status and determine factors that are associated with the duration from treatment initiation to death or treatment failure in children treated for DR-TB from February 2009 to February 2019 in Ethiopia.

## Methods

### Study setting and population

We conducted a retrospective cohort study in children younger than 15 years old who were treated for DR-TB since February 2009 to February 2019 at 41 DR-TB Treatment Initiating Centers (TICs) in Ethiopia. Programmatic management of DR-TB was started in February 2009 in Ethiopia [30]. Currently, there are two types of health facilities to treat DR-TB in the country. These are TICs and Treatment Follow up Centers (TFCs). The majority of DR-TB patients initiate their treatments in TICs while stable patients follow their scheduled SLDs under directly observed short course therapy programme in TFCs [32]. There are a total of 53 TICs and several TFCs to treat DR-TB patients in separate TB clinics in each health facility. However, all information on the patients enrolled into the DR-TB treatment programme has been documented at TICs. We included 41 TICs to this study, because the remaining 12 TICs had no patients who completed their treatment during the study period.

### Inclusion and exclusion criteria

We included children who were younger than 15 years old, diagnosed clinically or bacteriologically for DR-TB, and treated under the national TB programme from February 2009 to February 2019. The reason for enrolling children younger than 15 years old in this study was to be in line with WHO reporting method on age category and to address the existing limited evidence availability in this overlooked and at-risk population. However, we excluded three children whose final treatment outcome confirmation dates were missed. Bacteriologically confirmed DR-TB refers to those cases with documented laboratory DST results for drug resistance. Whereas, clinically diagnosed DR-TB refers to those cases with no documented DST results but the clinical panel team decided to treat the patients empirically with a course of treatment including SLDs based on clinical criteria and previous contact history [32].

### Diagnosis of drug resistant tuberculosis

WHO endorsed phenotypic and genotypic techniques were used to confirm DR-TB in Ethiopia. The phenotypic techniques include solid (Löwenstein-Jensen (LJ)) or liquid (BACTEC MGIT 960) culture media. The genotypic techniques are Line Probe Assay (LPA) and Xpert MTB/RIF assay. Xpert MTB/RIF assay is a rapid, sensitive and specific technique that has been under use to detect *M*. *tuberculosis* and rifampin resistant directly from the sputum, and it has been decentralized to the lower level health system in the country. Culture to isolate *M*. *tuberculosis* was performed at one national TB reference laboratory and nine regional reference laboratories based on the national TB diagnostic algorithm [32]. To diagnose pediatric DR-TB, the national treatment guideline recommends systematic contact tracing and screening of children at risk of DR-TB and monitoring response to the first line treatment in addition to the use of bacteriological methods [32].

### Drug resistant tuberculosis treatment

Previously, all DR-TB patients were being treated as inpatients for the first few months at treatment centers in Ethiopia [33]. However, with the recent edition of the national TB treatment guideline, all patients including children with DR-TB need to be treated under a clinic-based ambulatory model of care, unless the patients develop a severe adverse drug reaction during the course of the treatment [32]. Moreover, patients either with serious medical or social conditions could be admitted with the decision of the treatment panel [32]. The current DR-TB treatment regimens in Ethiopia consists of SLDs: levofloxacin, ethionamide, cycloserine, para-aminosalicyclic acid (PAS), pyrazinamide, prothionamide, linezolid, clofazimine and injectable drugs such as amikacin, kanamycin and capreomycin [32]. All the patients enrolled into this study were treated by a standardized long term regimen consists capreomycin, levofloxacin, prothionamide, cycloserine and high dose isonizid during the intensive phase. During the continuation phase, levofloxacin, prothionamide, cycloserine and high dose isonizid were used. Treatment with injectable drugs continues at least for eight months based on clinical, microbiological and radiographic evaluation outcomes [32]. The minimum treatment duration is 20 months which is at least 18 months after bacteriological conversion [32]. In Ethiopia, DR-TB treatment in children patients follows the basic principles of the regimen designed for use in adults, and they also receive a standardized long regimen [32]. All drugs are also dosed at the higher concentration of the recommended ranges, but cutting or crushing of pills into pieces would be required to achieve the recommended dose since most second line drugs do not have pediatric formulations. In addition, dosing of anti-TB drugs is calculated based on the current body weight of the child and adjusted regularly as weight changes during the treatment period. All doses are also administered on once-daily basis under strict supervision of a health-care worker. DR-TB treatment regimen in Ethiopia is the same over the last ten years with minor modification in recent version of treatment guideline. However, the long term standardized regimen is the same for all patients included in this study.

### Data collection

We collected data through reviewing clinical charts, registration books and laboratory reports. We collected data on socio-demographic variables such as sex, age and residence. We also collected data on clinical characteristics including previous TB treatment history, anatomical site of the disease, drug resistance type (MDR/RR-TB), drug resistance conformation method, HIV status, Antiretroviral Therapy (ART) status, weight and height of the children and adverse drug reactions. In addition, we collected information on baseline laboratory test results such as liver function tests (alanine transaminase (ALT), aspartate transaminase (AST)), renal function test (creatinine), hemoglobin level and white blood cell count. The national DR-TB treatment guideline recommends liver and renal function test per-month in intensive phase and as per-clinical indication in continuation phases for every patient [32]. The guideline also recommends hemoglobin examination at baseline and per-month for the patients on the regimen contains linezolid [32]. All data were collected by health professionals familiar with DR-TB treatment after two days of practical training on use of the data collection tool.

### Outcome variable

The main outcome variable in this study was the duration from treatment initiation to death or treatment failure, and it was collected from TB registration books. We adopted the final treatment outcome results of DR-TB in this study from WHO and national DR-TB treatment guidelines [32,34]. The final treatment outcome of DR-TB in this study is categorized into five groups for descriptive purpose: cured, treatment completed, treatment failed, lost to follow up and died. However, we considered treatment failure and death as failure event (failure) in survival analysis modeling, while cured, treatment completed and lost to follow up as censored. The definition of these treatment outcome categories are documented in national and WHO treatment guidelines [32,34]. For the children who were clinically diagnosed at enrollment, treatment failure was assessed by weight loss or clinical deterioration [32]. Thus, the treatment outcomes for children who did not gain weight or had shown clinical deterioration were assumed to be treatment failure.

### Data analysis

We entered data into CSPro software version 6.1 and performed data analysis using STATA version 14 (StataCorp, College Station, TX, USA). All data were confirmed and cleaned from data sources. We described participants’ demographic and clinical characteristics using descriptive statistics. We categorized age into two categories—younger than 5 years versus 5 to 14 years old. We also categorized hemoglobin level into four categories (normal, mild, moderate and severe) to indicate status of anemia based on WHO anemia categorization cut-off points [35]. Proportion of enrollment to the treatment was calculated by dividing the number of children receiving treatment by the total number of DR-TB patients treated during the specified years in the selected TICs. We used a multivariable Cox proportional hazard model to determine the independent effects of participants’ characteristics on death or treatment failure. The level of effects was reported by Hazard Ratio (HR) with 95% Confidence Intervals (CIs). We considered death and treatment failure as event of interest (failure) in Cox proportional hazard modeling. We included all variables that scored p-value less than 0.2 during a simple Cox proportional hazard analysis, and clinically or epidemiologically relevant variables into the multivariable model. We assessed the proportional hazard assumption of the Cox proportional hazard models before model fit and all variables included to the model were found to be meeting the assumption. A Kaplan-Meier survival curve was drawn for the variables independently associated with the failure. We set the level of significance at 5%.

### Ethical consideration

This study was approved by the research Ethics Review Board of Tehran University of Medical Sciences (approval number-IR.TUMS.SPH.REC.1396.4287), Ethiopian Public Health Institute (approval number-EPHI-IRB-065-2017), St. Peter’s Specialized Hospital (approval number-V81622018) and Armouer Hansen Research Institute (approval number-PO13/18). We also obtained a waiver of both informed consent and assent from each review board. Sensitive information that could identify patients was not reported in this article to assure confidentiality.

## Results

### Enrollment to the treatment

**Fig 1:**
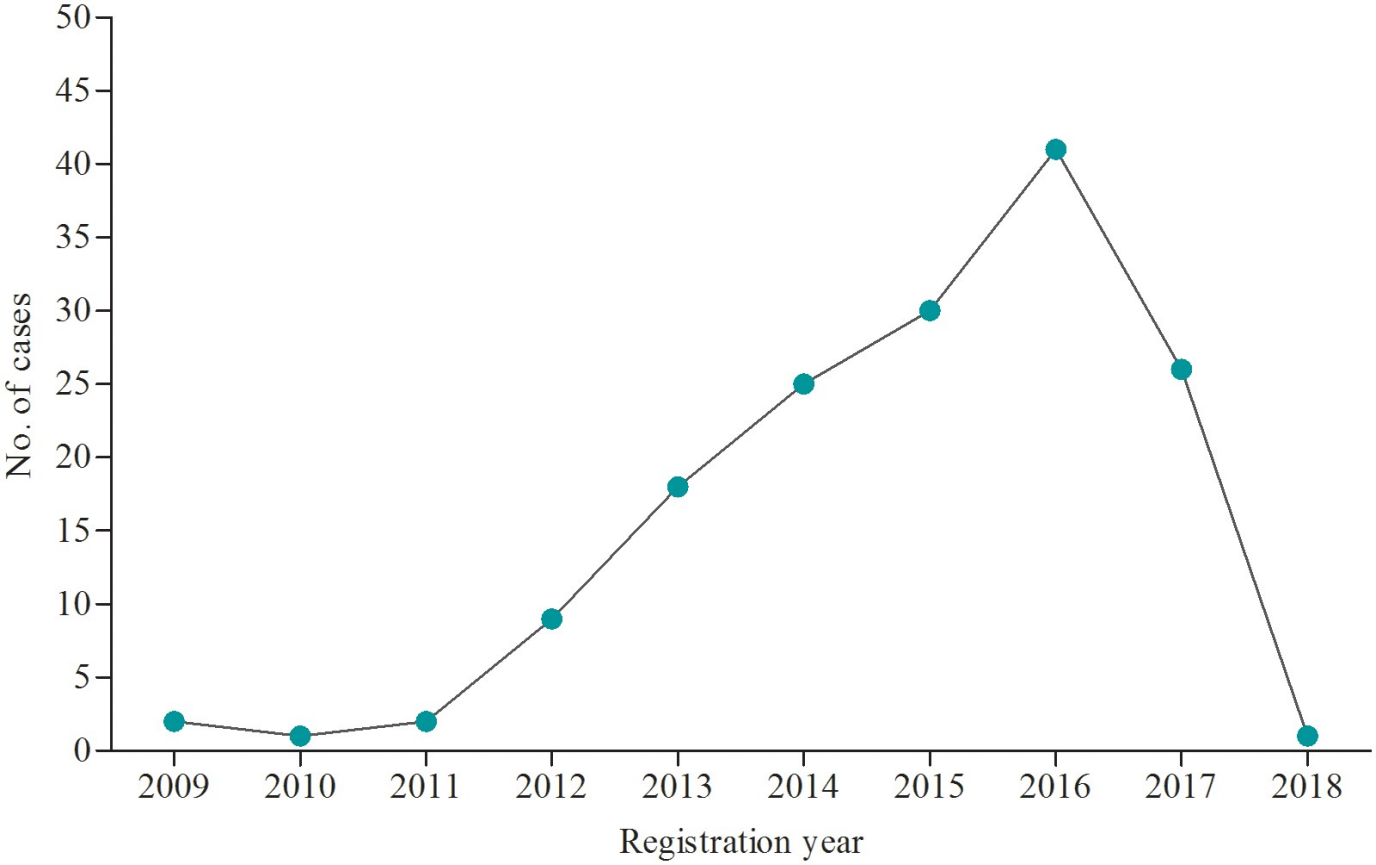
Drug resistant tuberculosis treatment enrollment trend in children, Ethiopia, 2009–2019.

A total of 3,478 DR-TB patients were registered for a treatment in 41 of 53 (77.4%) TICs in Ethiopia and of these, 155 (4.5%) were children younger than 15 years old. The final treatment outcome of all the 3,478 DR-TB patients were known by February 30, 2019. The TICs included in this study were reporting an average of six children with DR-TB over the last ten years. Fig 1 depicts enrollment trend of children with DR-TB in the last ten years. The highest pediatric DR-TB cases was registered and treated in the year 2016 with 41children whereas the lowest was in 2010 and 2018 with only one child [Fig 1].

### Participants’ characteristics

Table 1 shows characteristics of 155 children on DR-TB treatment in Ethiopia. Eighty eight (56.8%) of the patients were females, and the mean age was 9.2(SD ± 4.1) years with the age range of three months to 14 years. One hundred twenty nine (83.2%) of the children were above or equal to 5 years of age. One hundred one (65.2%) of the patients were infected with TB bacilli resistant to rifampin (isonizid susceptibility status unknown), and 117 (75.5%) patients were infected with pulmonary TB. Fifty six (36.1%) of the patients were treatment naïve. Resistance status of 72.3% isolates were bacteriologically confirmed [Table 1]. Of the 103 children whose previous DR-TB contact history was obtained, 44 (42.7%) had contact history. Furthermore, of the 155 children, 14 (9.0%) were HIV infected. Of these 14 children whose HIV sero-status were known, 12 (85.7%) were on ART. Seventy nine (71.8%) of children were hospitalized at the treatment initiation, and the mean duration of hospitalization among those who were hospitalized at the treatment initiation was 72.3 (±55.6) days. There was drug modification (cutting or crushing of pills into pieces) history for 11 (10.3%) children during the treatment period [Table 1].

**Table 1:** Demographic and clinical characteristics of the patients (n = 155 unless indicated).

Variable		n (%)
Sex	Male	67 (43.2)
	Female	88 (56.8)
Age	<5 years	26 (16.8)
	>= 5 years	129 (83.2)
Drug resistance type	RIF resistant/Isonizid susceptibility status unknown	101 (65.2)
	MDR-TB	54 (34.8)
Anatomical site of TB	Pulmonary	117 (75.5)
	Extra pulmonary	38 (24.5)
Previous TB treatment history	New	56 (36.1)
	Treatment after relapse	25 (16.1)
	Treatment after being lost to follow up	6 (3.9)
	Treatment after failure of new TB regimen	41 (26.5)
	Treatment after failure of retreatment regimen	27 (17.4)
Previous history of SLDs exposure (n = 132)	Yes	4 (3.0)
	No	128 (97.0)
Drug resistance diagnosis method	GeneXpert MTB/RIF	86 (55.5)
	Culture/LPA	26 (16.8)
	Clinical	43 (27.7)
Reasons for entering to DR-TB treatment	Bacteriologically confirmed	112 (72.3)
	Clinically diagnosed	43 (27.7)
HIV sero-status	Non-reactive	141 (91.0)
	Sero-reactive	14 (9.0)
ART status (n = 153)	Not applicable	141 (92.2)
	Started	12 (7.8)
DR-TB patient contact history (n = 103)	Yes	44 (42.7)
	No	59 (57.3)
Drug susceptible TB contact history (n = 80)	Yes	23 (28.8)
	No	57 (71.3)
	Unknown	
Hospitalization history at the beginning of treatment (n = 110)	Hospitalized	79 (71.8)
	Not hospitalized	31 (28.2)
History of drug modification during treatment period (n = 107)	Not modified	96 (89.7)
	Modified	11 (10.3)

TB-tuberculosis, ART-Antiretroviral therapy, SLDs-Second line drugs, HIV-Human immunodeficiency virus, DR-Multidrug resistant, LPA-Line probe Assay

Drug susceptibility testing was done for the four of the first-line drugs: rifampin, isonized, ethambutol and streptomycin [Table 2]. Rifampin susceptibility test was performed on 110 isolates and 98.2% demonstrated resistance to the therapy [Table 2]. The mean hemoglobin level was 12.4 (±2.1), and 39 (26.2%) of children had any grade of anemia at the treatment initiation.

**Table 2:** Anti-tuberculosis drug susceptibility test results.

Anti-tuberculosis drug	Susceptibility test results	n (%)
Rifampicin (n = 112)	Resistant	110 (98.2)
	Susceptible	2 (1.8)
Isonized (n = 60)	Resistant	56 (93.3)
	Susceptible	4 (6.7)
Ethambutol (n = 10)	Resistant	4 (40.0)
	Susceptible	6 (60)
Streptomycin (n = 11)	Resistant	6 (54.5)
	Susceptible	5 (45.5)

### Treatment outcome

**Fig 2:**
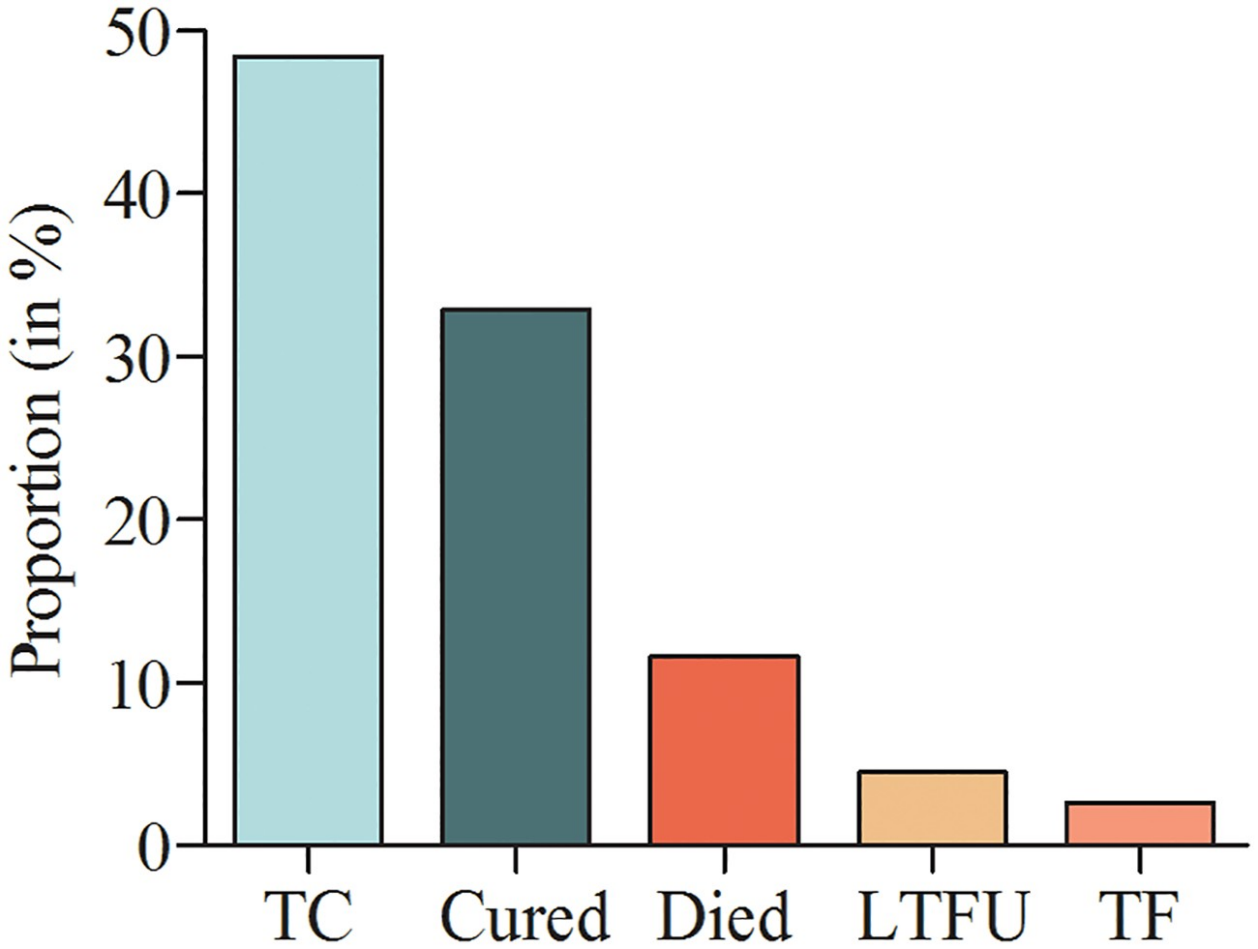
Treatment outcome among children on drug resistant TB treatment in Ethiopia, from 2009 to 2019 (TC-treatment completed, LTFU-lost to follow up, TF-treatment failed).

Fig 2 shows the treatment outcome proportion of 155 children who had final treatment outcome results by February 2019 in Ethiopia. Of 155 children with documented treatment outcome, 75 (48.4%) completed the treatment and 51 (32.9%) were cured [Fig 2]. Treatment success (cured plus treatment completed) was 126 (81.3%), and unsuccessful treatment outcome (treatment failed, lost to follow up and death) was 29 (18.7%).

### Factors associated with time to death or treatment failure

The overall proportion of failure event (i.e. treatment failure and death) was 22 (14.2%). We assessed the effect of sex, age category, previous TB treatment history, drug resistance type, HIV sero-status and anemia on the duration from treatment initiation to event (death or treatment failure). The hazard of death or treatment failure was significantly higher in children younger than 5 years compared to children with age ≥ 5 years [*AHR = 3*.*2 95% CI*, *(1*.*2 to 8*.*3); p = 0*.*017]*. HIV sero-reactive children were 5.3 more likely to be failed (death/treatment failure) earlier compared to non-reactive children *(p = 0*.*002)* [Table 3]. Similarly, children with any grade of anemia were 4.3 times more likely to be failed (death/treatment failure) earlier compared to non-anemic children *(p = 0*.*001)* [Table 3].

**Table 3:** Risk factors of death and treatment failure in children on DR-TB treatment in Ethiopia, 2009–2019.

Variable		UHR (95%CI)	P-value	AHR(95% CI)	P-value
Sex	Female	1.00			
	Male	1.9 (0.79–4.46)	0.153	1.8 (0.73–4.2)	0.207
Age category (year)	>= 5	1.00			
	<5	2.6 (1.1–6.6)	0.037	3.2 (1.2–8.3)	0.017
Anatomical sit of TB	EPTB	1.00			
	Pulmonary	7.2 (1.0–53.3)			
Previous treatment history	New	1.00			
	Previously treated	0.68 (0.28–1.6)	0.382		
Drug resistance type	Rifampcin resistant/Isonized susceptibility status unknown	1.00			
	MDR	1.3 (0.53–3.0)	0.382		
HIV sero-status	Non-reactive	1.00			
	Reactive	3.5 (1.3–9.6)	0.014	5.3 (1.8–14.9)	0.002
Hemoglobin	Normal	1.00			
	Ay grade of anaemia	3.9 (1.6–9.1)	0.002	4.3 (1.8–10.3)	0.001

HIV-Human immunodeficiency virus, UHR- Unadjusted hazard ratio, AHR- Adjusted hazard ratio CI-Confidence interval, DR-Multidrug resistant, EPTB- Extra pulmonary tuberculosis

**Fig 3:**
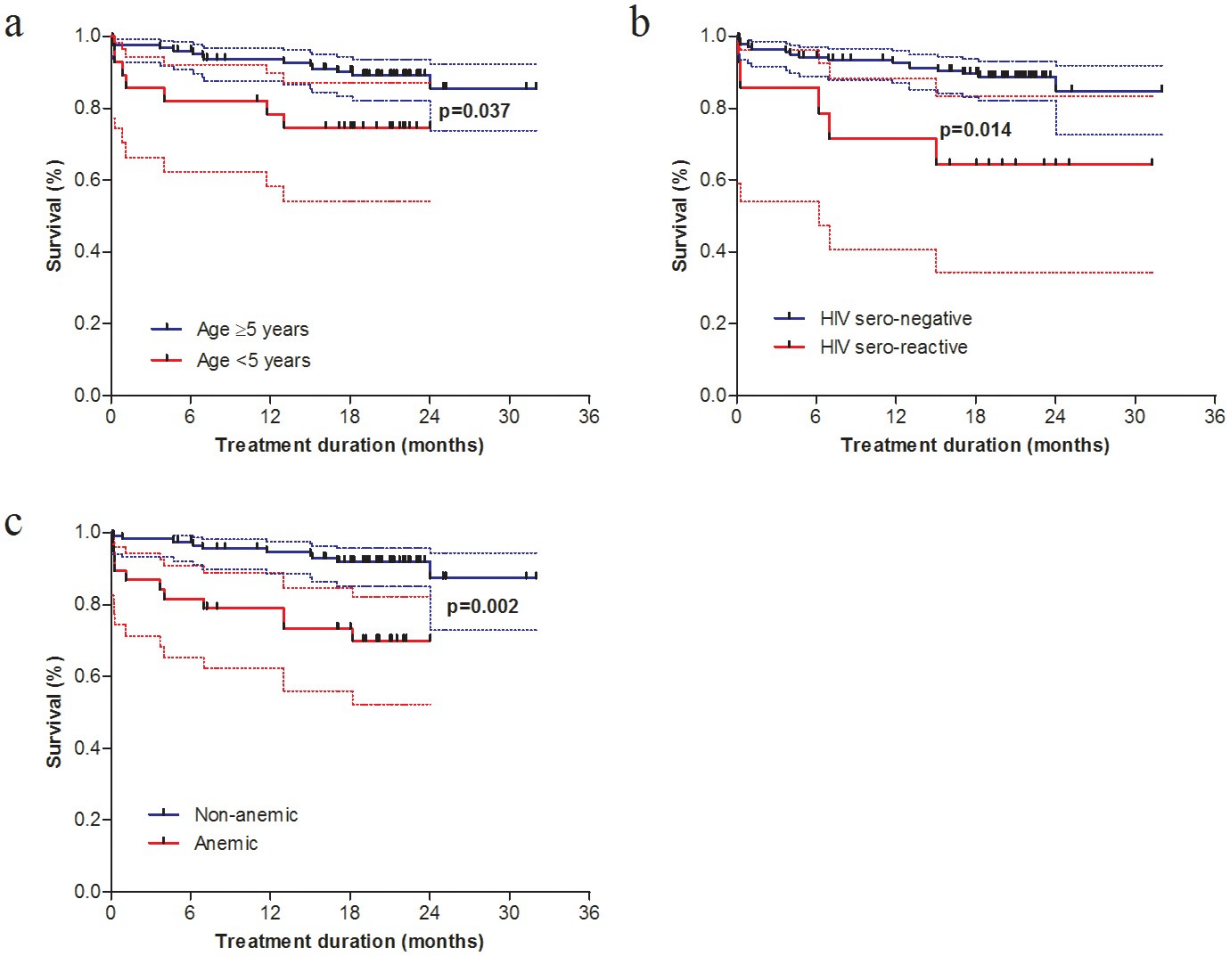
Kaplan-Meier survival curve for age category, HIV sero-status and hemoglobin category in children on DR-TB treatment (Dotted lines show 95% confidence interval).

Fig 3 shows a Kaplan-Meier survival analysis curve for the three variables (age category, HIV sero-status and anemia) that significantly predicted the duration from treatment initiation to death or treatment failure. A Kaplan-Meier survival curve indicated that the duration from the treatment initiation to failure (death or treatment failure) was higher in children younger than 5 years [Fig 3a; *p = 0*.*037*], being HIV sero-reactive [Fig 3b; *p = 0*.*014*] and with any grade of anemia [Fig 3c; *p = 0*.*002*].

## Discussion

The current study is a national level cohort study in children diagnosed and treated for DR-TB for a ten year period in Ethiopia. Of the 3,478 DR-TB patients who were diagnosed and enrolled into the treatment, only 155 (4.5%) were children younger than 15 years old. Furthermore, DR-TB treatment enrollment proportion in children was the lowest in the years 2017 and 2018. The overall treatment success in the children was 126 (81.3%), while the overall failure event (death and treatment failure considered as a failure event) was 22 (14.2%). In this cohort, the proportion of children who died (11.6%) was considerable. Hazard of death or treatment failure was higher in children younger than 5 years, HIV sero-reactive and in children with any grade of anemia.

Our findings indicated that the proportion of children diagnosed for DR-TB and enrolled into the treatment in Ethiopia in the last ten years is low compared to reports from elsewhere [11–13,16,17,19]. This implies that the majority of the deaths that occurred in children due to DR-TB could have been undiagnosed or untreated in the country. This low treatment enrollment of children infected with DR-TB in the country is due to poor laboratory infrastructures for DST, lack of clinical experience to detect the disease, poor health seeking behavior of the family and health system delays. The proportion of treatment enrollment in 2017 and 2018 was the lowest in this cohort. This could be due to the fact that the burden of DR-TB is decreasing in the country or probably emanating from case registration related problems due to the decentralization of treatment centers to the periphery.

Our study suggests that DR-TB treatment success rate (81.3%) in children who have received a standardized long regimen was as good as the treatment successes reported from other countries [11–13,16,17,19]. For instance in line with our finding, a review study that pooled data on 315 children from eight studies indicated an excellent treatment success rate (81.7%) [12]. In addition, a review study that pooled data on 975 children reported a 78% treatment success rate [13]. These review studies’ findings are in agreement with our finding. In contrast, previous studies from elsewhere in the world have shown low treatment success (ranging from 36% to 58%) in children on DR-TB treatment [15,18]. This discrepancy is most likely due to differences in sample size between the studies, severity of the disease, TB/HIV co-infection burden and treatment regimens. For example, unlike study reported by Hall et al [18] which was performed on a large sample size of patients (423), the sample size of the study conducted by Fairle et al [15] was only 13 children, which might underestimate the treatment success rate. In contrast, other studies even reported a higher proportion of treatment success rates than our finding [14,22]. This difference is due to the quality of care in the study area, disease severity status of the children, prevalence of TB/HIV co-infection and sample size difference. Interestingly, when we compare our finding with previously reported data on adult participants, our finding indicates that the DR-TB treatment success in children is relatively better than that for adults [28–30]. A study reported from Ethiopia on both children and adults also reported a relatively lower (78.6%) treatment success rate than our finding [30], which suggests that DR-TB treatment success in children is relatively better in children than in adults. In fact, in comparison to previously reported studies in adults, mortality seemed to be lower for children than for adults [12]. The probable explanation for the low mortality rate in children than in adults is due to the paucibacillary nature of the disease in children than in adults which leads to faster bacillary clearance in children [12,21].

The mortality in children in the current study was higher than previously reported studies from different countries [14,22,25], but it was similar to other studies [19,21,23]. Moreover, the proportion of children who died in our study was almost twice the result reported by a meta-analysis that pooled eight studies on 315 children (5.9%) [12]. This difference is due to the difference in the study period, quality of care and case registration, treatment regimens used, severity of the disease during diagnosis and nutritional status of the included children.

In agreement with our finding, a previous study also indicated that HIV sero-reactive significantly predicted death in children treated for DR-TB [23]. Moreover, a study by Hall et al [18] indicated that the mortality rate is twofold higher in children who were HIV sero-reactive than those who were non-reactive. The findings of these two studies are in line with our finding in which the hazard of death or treatment failure was 5.3 times greater in HIV sero-reactive children than those who were HIV non-reactive. Moreover, a study conducted on both adults and children in Ethiopia indicated that HIV sero-reactive significantly predicted the duration from treatment beginning to treatment failure/death [31]. However, the earlier study reported from Ethiopia has included only 5% of patients who were younger than 18 years old [31]. The majority of the children co-infected with HIV in our study (12/14 = 85.7%) were on ART. In any case the significant effect of HIV sero-reactivity on treatment outcome might be due to low CD4 count, high viral load and severity of the disease at enrollment. Since data on CD4 count, HIV viral load level and disease severity status at enrollment were not registered in our data sources, we could not assess their effect on the treatment outcome.

Age younger than five years was also significantly associated with the death or treatment failure in this study. In contrast, a previous study indicates that older children are more likely to die than the younger ones [25]. This difference is due to the variation in severity of the disease at treatment initiation, treatment adherence and TB/HIV co-infection burden in the study area. In agreement with our finding, evidence indicated that immature immune system at younger age leads to poor treatment outcome [36,37].

The presence of any grade of anemia was independently associated with the duration from treatment initiation to death or treatment failure in the current study. We could not find any report that indicated the effect of anemia on the treatment outcome of DR-TB in children. However, the study reported from Ethiopia on both adults and children indicated that anemia was independently associated with time to poor treatment outcome and the hazard of poor treatment outcome was 4.2 times higher in anemic patients than non-anemic [31]. This result was similar with our finding in which the hazard of death or treatment failure was 4.3 times higher in the children with any grade of anemia than those who were non-anemic. The presence of anemia in the children at the treatment initiation might be due to malnutrition or parasitic infection.

The main strength of our study was the inclusion of relatively large number of participants, and being the first report on treatment outcome status of children with DR-TB in Ethiopia at national and regional levels. Nevertheless, this study had also limitations. As the data of this study was collected retrospectively from routine DR-TB treatment programme, the main limitation of this study was that some variables were missing. Data on many laboratory tests, weight and height of the children and adverse drug reactions were not recoded and several were missing. Moreover, due to poor record of registration and lack of laboratory results during the follow up period, there was no follow up data on treatment monitoring laboratory tests for almost all children. There were also data missing for liver and renal function enzymes tests for a considerable number of children and we obtained only baseline hemoglobin results for the included children. Thus, missing data on key variables limited our analysis to determine factors associated with duration from treatment initiation to death or treatment failure at baseline and during follow up period. In addition, apart from rifampin, there were few DST results for other first line and all SLDs for many children. This also hindered the explanation of drug susceptibility patterns and their effects on treatment outcome in children with DR-TB.

## Conclusion

In this study, the proportion of children enrolled to DR-TB treatment was lower than the proportion of adults enrolled to the DR-TB treatment (4.5% in children versus 95.5% in adults). Our findings also suggest that children with DR-TB can be successfully treated with a standardized long regimen in a resource limited and high TB/HIV burden settings. The overall proportion of children who achieved treatment success in this study was relatively higher than the findings of the previously conducted studies on adults from Ethiopia. The hazard of death or treatment failure was significantly higher in children who were younger than 5 years old, HIV sero-reactive and in children with any grade of anemia. Further prospective cohort study is required to investigate factors contributing to the duration from the treatment initiation to death or treatment failure and low proportion of treatment enrollment. Most importantly, targeting younger children, those who are HIV sero-reactive and anemic is vital to improve DR-TB treatment success.

## Supporting information

S1 Dataset (SAV)
